# Effects of addition of *Trichoderma* spp. on growth and soil microorganisms of continuously cropped *Salvia miltiorrhiza*

**DOI:** 10.3389/fpls.2025.1654385

**Published:** 2025-09-03

**Authors:** Linlin Zhang, Xiaoxi Pan, Yue Zhang, Yiming Guan, Yincun Li, Qiao Jin, Keming Cao, Chenggang Shan, Qiuxia Wang

**Affiliations:** ^1^ Institute of Special Animal and Plant Sciences, Chinese Academy of Agricultural Sciences, Changchun, China; ^2^ Institute of Industrial Crops, Shandong Academy of Agricultural Sciences, Jinan, China; ^3^ Jilin Provincial Key Laboratory of Traditional Chinese Medicinal Materials Cultivation and Propagation, Changchun, China

**Keywords:** *Trichoderma* spp., *Salvia miltiorrhiza*, soil nutrient, microorganism, effective ingredient

## Abstract

**Introduction:**

Continuous cropping (CC) poses significant challenges to the yield and quality of Salvia miltiorrhiza, a medicinally important plant with annual market demand of around 20 million kg. Previous studies have explored chemical mitigation methods, but concerns persist regarding environmental pollution and safety issues.

**Methods:**

In this study, we evaluated three strains of the fungus *Trichoderma* (*T. brevicompactum*, *T. viridescens*, and *T. velutinum*) as ecofriendly alternatives for CC obstacle mitigation in *S. miltiorrhiza* cultivation.

**Results:**

The *Trichoderma* treatments significantly enhanced the relative abundance of beneficial soil bacteria (Actinobacteria 9.25%–16.88%; Chloroflexi 6.41%–16.73%; Gemmatimonadetes 1.21%–3.16%), while decreasing the abundance of pathogenic *Fusarium* by 12.67%–31.75%. Soil analysis revealed substantial improvements in total organic carbon (47.35%–65.88%), nitrate nitrogen (91.38%–318.89%), available potassium (4.29%–17.16%), and available phosphorus (1.85%–11.86%) following *Trichoderma* treatment. *T. brevicompactum* demonstrated superior performance among the three tested strains, increasing the individual plant fresh weight by 17.79% and the survival rate by 13.33%. This treatment also significantly elevated (p < 0.05) the content of key bioactive compounds in *S. miltiorrhiza* root: tanshinone IIA (51.16% increase), cryptotanshinone (56.76%), tanshinone I (50.00%), and salvianolic acid B (18.43%).

**Discussion:**

Trichoderma can effectively alleviate *S. miltiorrhiza* CC obstacles by improving the soil nutrient status and modulating the soil microbial community, thereby enhancing plant growth and stress resistance. This study provides a promising ecofriendly strategy for sustainable cultivation of *S. miltiorrhiza* and other medicinal plants facing similar challenges.

## 1 Introduction

*Salvia miltiorrhiza* is a perennial plant native to China and Japan, also known as red sage or Chinese sage. Its roots are a commonly used bulk medicinal material, which has demonstrated therapeutic efficacy in the treatment of pathologies including cardiovascular and cerebrovascular diseases, liver diseases, and diabetes (Feng et al., 2022; Zhang et al., 2024c). The annual market demand for *S. miltiorrhiza* is around 20 million kg, and this is increasing (Liu et al., 2020). With the increase in the planting area of *S. miltiorrhiza*, the problem of continuous cropping (CC) obstacles has gradually become prominent.

CC obstacles are a prevalent issue in cultivation of medicinal plants (Zeeshan Ul. Haq. et al., 2023), particularly for root-derived materials (Yang et al., 2024b). CC compromises plant growth because of deteriorating soil physicochemical properties, disrupted soil microbial communities, and increased soil-borne diseases, leading to decreased crop productivity and quality (Chen et al., 2022; Su et al., 2023). Previous studies demonstrated that the incidence of root diseases in *S. miltiorrhiza* reached approximately 10% after one year of cultivation, escalating to 60%–70% following three consecutive cropping cycles (Pu et al., 2022; Ju et al., 2023). Furthermore, excessive dependence on chemical fertilizers and pesticides has been documented to exacerbate soil degradation and lead to accumulation of pesticide residues (Yan et al., 2022). Consequently, CC has emerged as a critical constraint on sustainable *S. miltiorrhiza* production. Although preliminary insights into the CC obstacle mechanisms have been obtained, effective management strategies remain exploratory due to system complexity.

Previous studies have demonstrated that dazomet and chloropicrin fumigation can enhance the yield of crops and reduce the abundance of harmful microorganisms (Wang et al., 2023; Chen et al., 2023). However, the application of chemical methods has potential risks, including environmental pollution, ecosystem disruption, and consumer safety concerns. In contrast, research has confirmed that biocontrol microorganisms (e.g., *Trichoderma* spp.) promote plant biomass by modifying soil conditions (Meng et al., 2019; Wang et al., 2021), offering a nontoxic, ecofriendly alternative without chemical residues.

*Trichoderma* spp. are beneficial fungi in soil and plant root ecosystems (Kashyap et al., 2017). Numerous studies have shown that *Trichoderma* spp. have significant control effects on various plant diseases (Rees et al., 2022; Rodrigues et al., 2023), and promote plant growth and improve soil health (Wu et al., 2022; Zhang et al., 2024b). *Trichoderma* has been demonstrated to alleviate CC obstacles. Chen et al (2011, 2012) found that application of *T. harzianum* effectively degraded allelopathic substances in the rhizosphere of continuously cropped cucumber, diversified the soil microbial community, and controlled cucumber wilt disease. Similarly, Mao and Jiang (2021) reported that application of *Trichoderma* MHT1134 significantly decreased the incidence of *Capsicum* wilt and improved the soil microbiome composition in CC conditions. In five-year continuously cropped watermelon soil, *T. asperellum* M45a granules improved *Fusarium* wilt control efficacy by up to 67.44% during flowering and significantly enhanced soil nutrient conversion rates [total nitrogen (TN), nitrate-nitrogen (NO_3_^—^N), and available phosphorus (AP)] (Zhang et al., 2020). However, the effects of *Trichoderma* on *S. miltiorrhiza* remain unexplored.

In this study, three soil-isolated *Trichoderma* strains were selected to investigate their impacts on biomass, soil chemical properties, and microbial community structure for continuously cropped *S. miltiorrhiza*. Our findings may provide valuable insights for sustainable, pollution-free *S. miltiorrhiza* production.

## 2 Materials and methods

### 2.1 Fungal strains

Three *Trichoderma* strains were obtained from the Institute of Special Wild Economic Animal and Plant Sciences, Chinese Academy of Agricultural Sciences, Changchun, China. They were isolated from ginseng roots in samples from Zuojia Town (Jilin City, Jilin Province, China). Following morphological and molecular analyses, these strains were identified as *T. brevicompactum* (CGMCC No. 23213), *T. viridescens* (CGMCC No. 23212), and *T. velutinum* (CGMCC No. 23211), and preserved at the China General Microbiological Culture Collection Center (CGMCC).

### 2.2 Experimental design

This study was carried out between May and November 2022 in Pingyi County, Linyi City, Shandong Province, China. This plot has been continuously planted with *S. miltiorrhiza* for 6 years. The physicochemical properties of the foundation soil are presented in **Table 1**. The experiments consisted of four treatments: *T. brevicompactum* (A); *T. viridescens* (B); *T. velutinum* (C); and untreated plants as control CK (D), with a plot area of 6 m^2^ for each treatment. Each treatment had three replicates, with 150 seedlings per plot. *S. miltiorrhiza* seedlings with consistent growth and plant height were selected for transplantation.

**Table 1:** Effects of different treatments on soil nutrients (mean ± SE).

Soil properties	CK	A	B	C	FS
pH	5.91± 0.06a	5.72 ± 0.05b	5.66 ± 0.04b	5.70± 0.06b	6.13 ± 0.06
TN (g/kg)	0.19 ± 0.02c	0.62± 0.03a	0.58 ± 0.06ab	0.49± 0.01b	0.46 ± 0.05
TOC (g/kg)	3.4± 0.35b	5.64 ± 0.21a	5.56± 0.48a	5.01± 0.33a	4.66 ± 0.33
NH_4_^+^-N (mg/kg)	8.7± 0.25c	13.45 ± 0.11a	2.95± 1.07d	11.40± 0.56b	9.35 ± 0.35
NO_3_^−^-N (mg/kg)	0.58± 0.08a	2.43± 0.40a	1.11± 0.02a	1.93 ± 1.01a	9.17 ± 0.24
AP (mg/kg)	46.37 ± 8.75a	47.23 ± 3.4a	51.87 ± 0.98a	51.73 ± 0.67a	39.25 ± 0.43
AK (mg/kg)	62.01 ± 6.91a	64.67 ± 5.80a	69.99 ± 6.91a	72.65 ± 16.18a	49.89 ± 5.53

CK, no treatment; A, treatment with *T. brevicompactum*; B, treatment with *T. velutinum*; C, treatment with *T. viridescens*; FS, foundation soil. TN, Total nitrogen; TOC, total soil organic carbon; NH_4_^+^-N, ammonium nitrogen; NO_3_^−^-N, nitrate-nitrogen; AP, available phosphorus; AK, available potassium. Different lowercase letters within a row indicate statistically significant differences (*p*< 0.05).

*Trichoderma* strain was inoculated on PDA medium and cultured at 25°C for 7 days, add 10 ml of sterile water to the petri dish 7 days after inoculation. The surface of the medium was gently scraped with an inoculation needle, and then the spore suspension was prepared after gauze filtration, precipitation and sterile water suspension. After transplantation, the treatment group was irrigated with a *Trichoderma* spore suspension (10^6^ colony-forming units/ml) at a dosage of 30 ml per plant, once every 20 days, for a total of three times. The roots of the CK group were irrigated with the same amount of water per plant. Soil samples were obtained at the termination of the growth period of *S. miltiorrhiza*. Rhizosphere soil and bulk soil samples were collected in sterile plastic containers. Portions of each sample were preserved at −80°C for microbial DNA analysis, with the remainder air-dried for assessment of chemical properties. The fresh weight of whole *S. miltiorrhiza* plants was recorded after complete cleaning. Plant dry weight was measured after desiccation at 105°C to constant mass.

The *T. brevicompactum* strain that had the best comprehensive effect was made into a microbial preparation. It was applied in Pingyi County, Linyi City, Shandong Province, from May 2024 to November 2024. These experiments included two treatments: PA (*T. brevicompactum*) and PCK (untreated plants, control). After one growing season, *S. miltiorrhiza* samples were collected and determined for fresh weight, survival rate, and effective ingredient content.

### 2.3 Determination of soil chemical properties

Soil pH was measured in soil–water suspensions (1:2.5) using a pH meter. Total nitrogen (TN) and total soil organic carbon (TOC) contents were determined using an elemental analyzer (Vario EL, Germany). Ammonium nitrogen (NH_4_^+^-N) and nitrate-nitrogen (NO_3_^−^-N) contents in the soil were analyzed with a continuous flow analyzer (SEAL AA3, Germany) (Jin et al., 2022). The available phosphorus (AP) content was measured by performing lixiviating-molybdenum blue colorimetry after extraction with 0.5 M NaHCO_3_ for 30 min. The available potassium (AK) content was measured using the NH_4_OAc extraction-flame spectrophotometer method (Bao, 2000).

### 2.4 Soil DNA extraction, polymerase chain reaction, and sequencing

DNA extraction was conducted using a PowerSoil DNA Isolation Kit (Mo Bio Laboratories, Carlsbad, CA, USA). The bacterial 16S rRNA gene was amplified using primers 806R (GGACTACHVGGGTWTCTAAT) and 338F (ACTCCTACGGGAGGCAGCA), as described by Fu et al. (2023). PCR conditions: initial denaturation at 95°C (10 min), 40 amplification cycles (95°C for 15 s, 55°C for 60 s, and 72°C for 90 s), and a final extension at 72°C (7 min). The fungal internal transcribed spacer (ITS) region was amplified with primers ITS1F/ITS2 (CTTGGTCATTTAGAGGAAGTAA/GCTGCGTTCTTCATCGATGC) in conditions: initial denaturation at 95°C (5 min), and 35 amplification cycles (95°C for 1 min, 53°C for 45 s, and 72°C for 1 min). The amplicons were purified and sequenced on an Illumina MiSeq platform (Illumina Inc., San Diego, CA, USA). The raw sequencing data generated in this study have been deposited in the China National Center for Bioinformation (https://bigd.big.ac.cn/gsa/browse/CRA025401).

Sequence data were processed using Trimmomatic v0.33 for quality filtering and Cutadapt v1.9.1 for primer removal (Martin, 2011; Bolger et al., 2014). Merged reads were generated using USEARCH v10 (Edgar, 2013), followed by chimera removal with UCHIME v4.2 (Edgar et al., 2011) to obtain quality-filtered sequences. Sequences were clustered into operational taxonomic units (OTUs) by 97% similarity using USEARCH. OTUs with total counts below two were excluded from further analysis. Taxonomy annotation was performed using the Naive Bayes classifier (70% confidence threshold) in QIIME2 with the SILVA database (release 138.1) as the reference (Quast et al., 2013; Bolyen et al., 2019).

### 2.5 Analysis of effective ingredient content

Quantitative determination of bioactive constituents in *S. miltiorrhiza* root, including of tanshinones (cryptotanshinone, tanshinone I, and tanshinone IIA) and salvianolic acid B, was performed using high-performance liquid chromatography (HPLC). For the tanshinones, the chromatographic conditions involved separation in octadecylsilane-bonded silica gel using acetonitrile–0.02% phosphoric acid gradient elution (20°C) with detection at 270 nm. The theoretical plate number should not be less than 60,000, calculated based on the tanshinone IIA peak. Reference and test solutions were prepared, with the latter obtained by precisely weighing about 0.3 g of powdered sample, extracting with 50 mL of methanol via ultrasonication, and adjusting for the weight loss with more methanol. Both solutions were subjected to HPLC analysis. The relative retention times of cryptotanshinone and tanshinone I, referenced to tanshinone IIA, must be within ±5% of the specified values; their contents were calculated using the peak area of tanshinone IIA and respective correction factors.

Salvianolic acid B was analyzed in modified conditions: acetonitrile–0.1% phosphoric acid (22:78 v:v) at a flow-rate of 1.2 mL/min with detection at 286 nm. The theoretical plate number should not be less than 6,000, calculated based on the salvianolic acid B peak. Salvianolic acid B reference solution (0.10 mg/mL) was prepared in methanol–water (8:2 v:v). The test solution was prepared similarly but with 0.15 g of powdered sample and 50 mL of the methanol–water mixture. Both solutions were analyzed by HPLC in the established chromatographic conditions.

### 2.6 Statistical analysis

Statistical comparisons were performed using one-way analysis of variance followed by the least significance difference *post-hoc* test (*p* < 0.05) to compare the mean values of samples, with data variability expressed as standard error (*n* = 3). Redundancy analysis (RDA) was conducted using the vegan package (v2.6-8) in R software (v4.4.1). Pearson correlation analyses were implemented with the psych package (v2.4.6.26).

## 3 Results

### 3.1 Comparison of the fresh and dry weights of *S. miltiorrhiza* in different treatments

**Figure 1:**
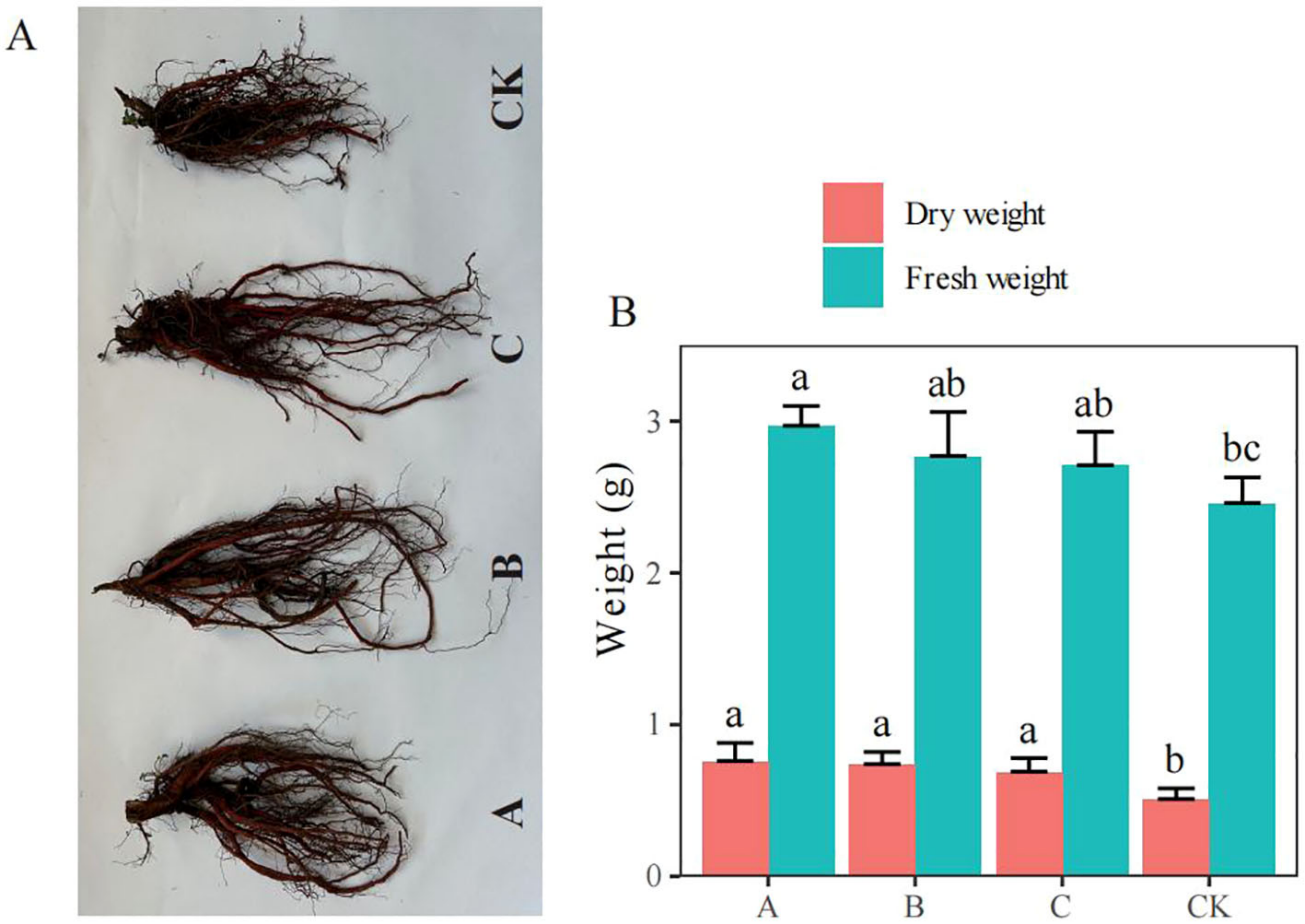
Effects of *Trichoderma* treatment on *Salvia miltiorrhiza* biomass production (mean ± SE). **(A)** Photographs of *S. miltiorrhiza*; **(B)** Fresh and dry weights of *S. miltiorrhiza*. CK, no treatment; A, *T. brevicompactum* treatment; B, *T. velutinum* treatment; C, *T. viridescens* treatment. Different lowercase letters above columns indicate significant differences (*p* < 0.05).

Compared with the fresh and dry weights of *S. miltiorrhiza* in the control (CK) group, those of *S. miltiorrhiza* in the A treatment group (*T. brevicompactum*) were increased by 20.73% and 49.02% (*p*< 0.05), respectively; those of *S. miltiorrhiza* in the B treatment group (*T. viridescens*) were increased by 12.60% and 45.10% (*p* < 0.05), respectively; and those of *S. miltiorrhiza* in the C treatment group (*T. velutinum*) were increased by 10.16% and 35.29% (*p* < 0.05), respectively (**Figure 1**). All the treatments markedly increased the dry weight, and treatment A substantially increased the fresh weight.

### 3.2 Effects of different treatments on soil chemical properties

The soil chemical properties in the *Trichoderma* treatment groups are shown in **Table 1**. Compared with the control (CK) group, the *Trichoderma* treatment groups notably decreased the soil pH value, and remarkably increased the TN and TOC contents, by 157.89%–226.32% and 47.35%–65.88%, respectively (*p* < 0.05). Treatments A and C significantly enhanced the NH_4_^+^-N content relative to the CK group (*p* < 0.05). *Trichoderma* treatment increased NO_3_^-^-N levels by 91.38%–318.89%, AP by 1.85%–11.86%, and AK by 4.29%–17.16% relative to the CK group.

### 3.3 Variations in soil microbial community composition across treatment groups

#### 3.3.1 Bacterial and fungal community structures at the phylum level

**Figure 2:**
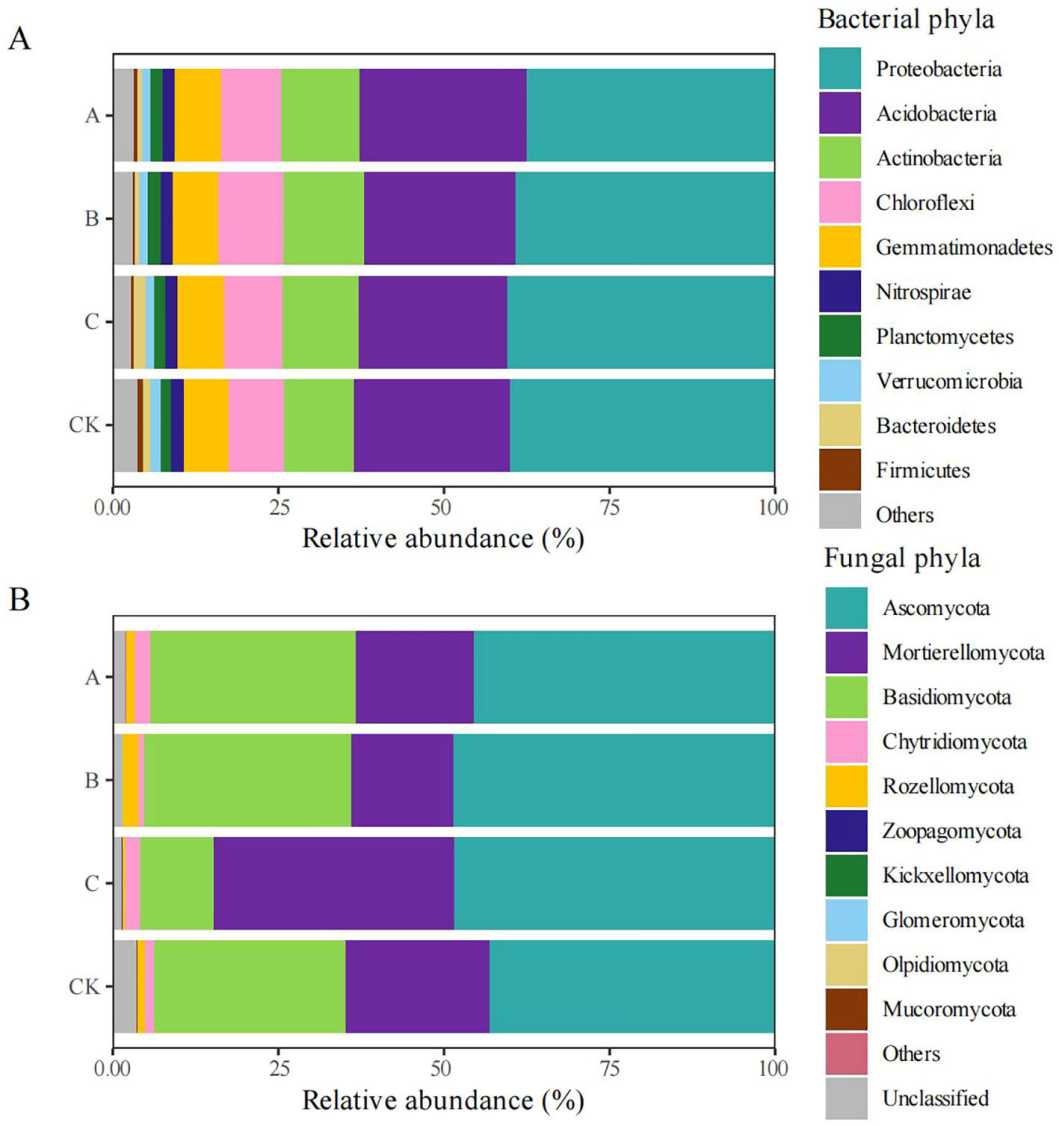
Relative abundance (%) of major bacterial and fungal phyla among treatment groups. The phylum abundance of bacteria **(A)** and fungi **(B)** in different groups: CK, no treatment; A, *T. brevicompactum* treatment; B, *T. velutinum* treatment; C, *T. viridescens* treatment.

No significant differences were observed between treatments in the relative abundances of the top 10 bacterial phyla, including Proteobacteria, Acidobacteria, Actinobacteria, Chloroflexi, Gemmatimonadetes, Nitrospirae, Planctomycetes, Verrucomicrobia, Bacteroidetes, and Firmicutes. However, quantitative analysis revealed subtle differences in relative abundances between treatments. The three dominant phyla (Proteobacteria, Acidobacteria, and Actinobacteria) represented 74.32%–74.55% of the total bacterial composition (**Figure 2A**). Compared with the CK group, the relative abundances of Actinobacteria, Chloroflexi, and Gemmatimonadetes were increased in the *Trichoderma* treatment groups. The maximum relative abundances of Actinobacteria and Chloroflexi occurred in treatment B (*T. viridescens*), while treatment A (*T. brevicompactum*) contained the highest proportion of Gemmatimonadetes.

The fungal community composition showed no significant variations in the top 10 phyla between treatments; these phyla were Ascomycota, Mortierellomycota, Basidiomycota, Chytridiomycota, Rozellomycota, Zoopagomycota, Kickxellomycota, Glomeromycota, Olpidiomycota, and Mucoromycota. However, the relative abundances of phyla exhibited some differences between treatments. Ascomycota, Mortierellomycota, and Basidiomycota collectively represented 94.41%–95.93% of fungal sequences (**Figure 2B**). The relative abundance of Ascomycota was significantly higher in the *Trichoderma*-treated groups than in the control (CK), reaching its maximum in treatment B (*T. viridescens*). *Trichoderma* inoculation suppressed Olpidiomycota and Mucoromycota populations, showing maximal suppression in treatment C (*T. velutinum*).

#### 3.3.2 Classification of bacteria and fungi at the genus level

**Figure 3:**
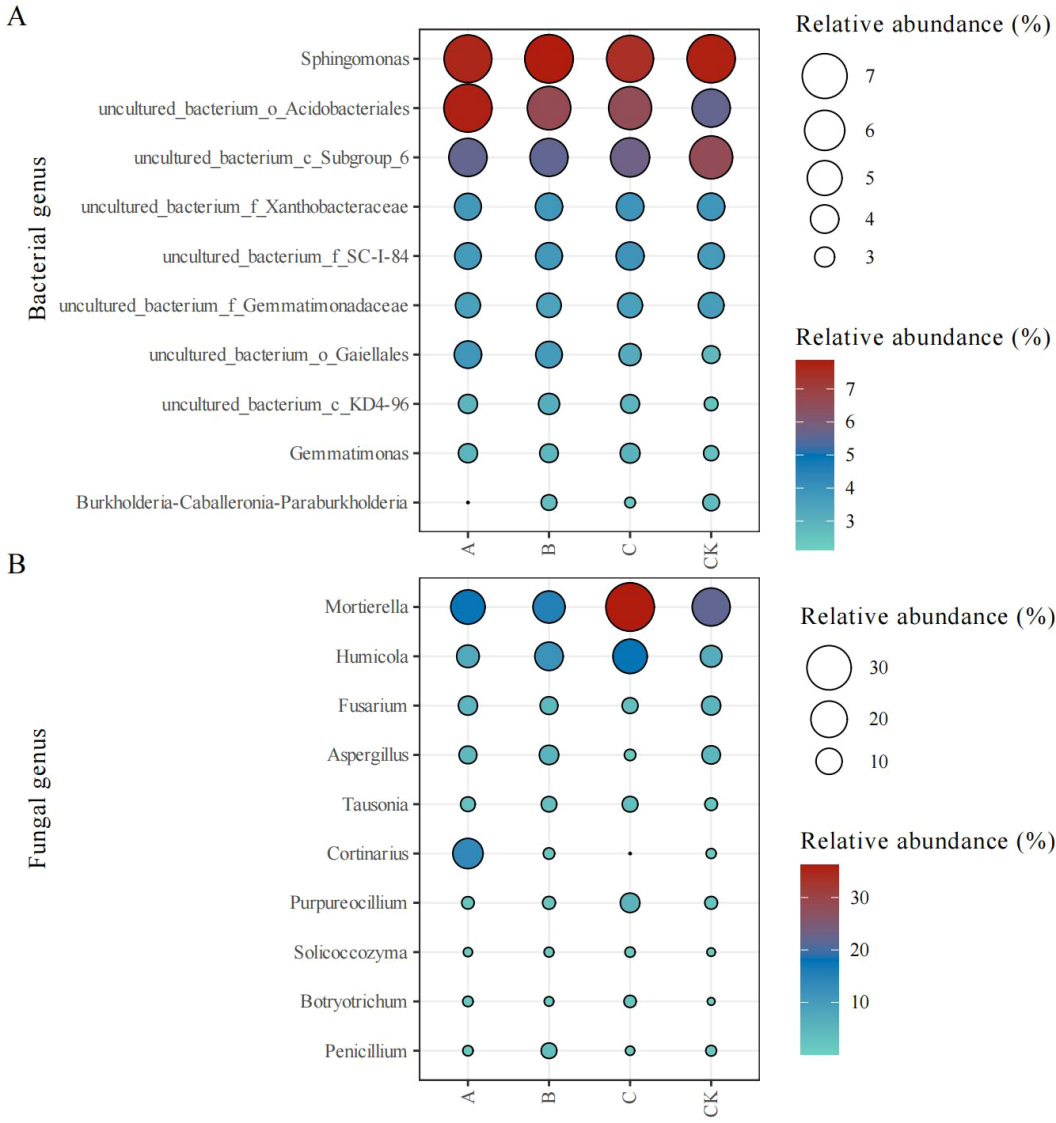
Relative abundance (%) of bacterial **(A)** and fungal **(B)** genera in different treatment groups. CK, no treatment; A, *T. brevicompactum* treatment; B, *T. velutinum* treatment; C, *T. viridescens* treatment.

The top 10 bacterial genera showed stable relative abundances across treatments (**Figure 3A**); however, minor variations in relative abundance were observed. Trichoderma application enhanced the abundance of *Gemmatimonas*, uncultured_bacterium_o_Acidobacteriales, uncultured_bacterium_f_SC-I-84, uncultured_bacterium_o_Gaiellales, and uncultured_bacterium_c_KD4-96, compared with the control (CK) treatment.

The composition of the top 10 fungal genera was slightly different between treatment groups (**Figure 3B**). The *Trichoderma* treatment groups showed an increased relative abundance of *Humicola*, *Tausonia*, *Solicoccozyma*, and *Botryotrichum* and a decreased relative abundance of *Fusarium* relative to the CK group.

### 3.4 Redundancy analysis of microbial communities and nutrients in the soil under different treatments

**Figure 4:**
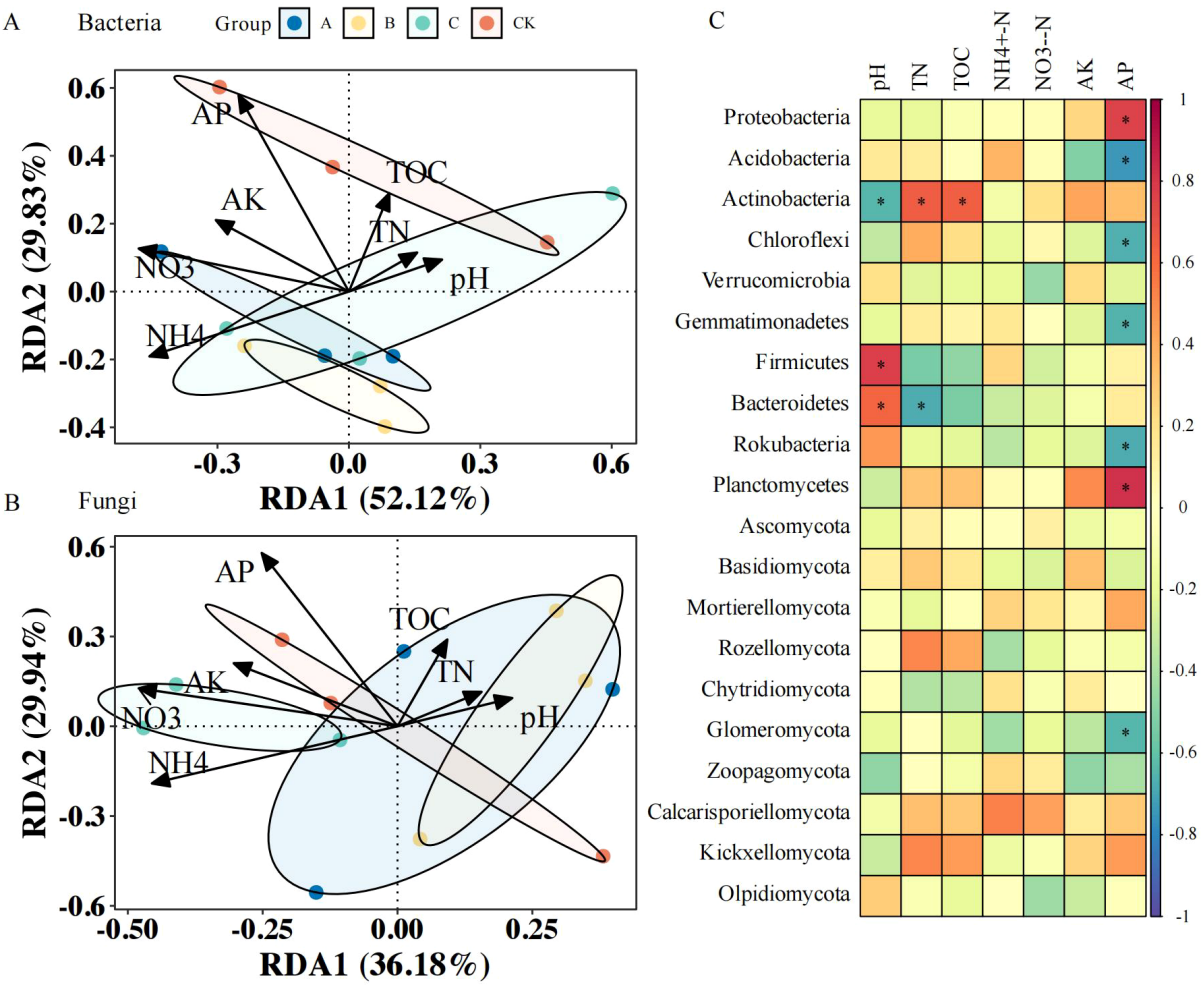
Soil nutrient–microbiome interactions in different treatments. **(A)** Redundancy analysis (RDA) of soil nutrients and bacterial community; **(B)** RDA of soil nutrients and fungal community; **(C)** Pearson’s correlation analysis of soil nutrients and bacterial and fungal phyla. CK, no treatment; A, *T. brevicompactum* treatment; B, *T. velutinum* treatment; C, *T. viridescens* treatment; TN, Total nitrogen; TOC, total soil organic carbon; NH_4_^+^-N, ammonium nitrogen; NO_3_^−^-N, nitrate-nitrogen; AP, available phosphorus; AK, available potassium. * provided p<0.05.

Redundancy analysis illustrated associations between the soil bacterial community composition and physicochemical parameters; RDA axes 1 and 2 explained 52.12% and 29.83% of the total variance respectively, cumulatively accounting for 81.95% of the variation in the dataset (**Figure 4A**). AP, NO_3_^−^-N, and NH_4_^+^-N were the main physicochemical factors associated with soil bacterial communities. As indicated in **Figure 4C**, AP correlated positively with Proteobacteria and Planctomycetes, but negatively with Acidobacteria, Chloroflexi, Gemmatimonadetes, and Rokubacteria (*p* < 0.05); the soil pH was positively related to Firmicutes and Bacteroidetes, and negatively related to Actinobacteria (*p* < 0.05); TN was positively associated with Actinobacteria and negatively associated with Bacteroidetes (*p* < 0.05); TOC content showed a strong positive association with the abundance of Actinobacteria (*p* < 0.05).

The soil fungal communities also showed significant covariation with physicochemical properties in RDA (**Figure 4B**). The RDA ordination explained 66.12% of total variance through axes 1 and 2 (36.18% and 29.94% respectively). AP, NO_3_^—^N, and NH_4_^+^-N were the main physicochemical factors associated with the soil fungal community. As shown in **Figure 4B**, the AP content was significantly negatively associated with Glomeromycota (*p* < 0.05).

### 3.5 Effect of *T. brevicompactum* application on secondary metabolite production in *S. miltiorrhiza*

Based on the experiments above, *T. brevicompactum* was identified as the most effective agent for improving *S. miltiorrhiza* cultivation. A further set of experiments was then conducted in which *T. brevicompactum* was applied to *S. miltiorrhiza* cultivation.

**Figure 5:**
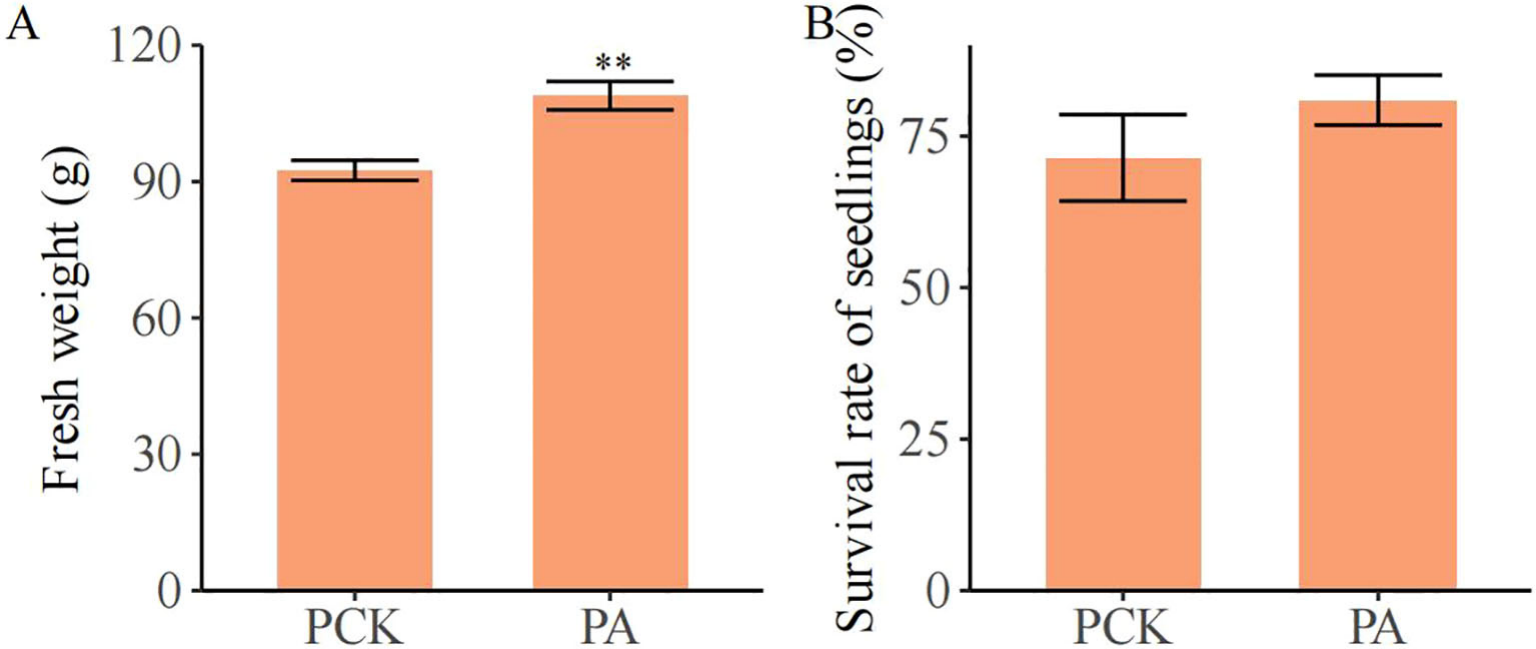
The effect of *T. brevicompactum* on **(A)** the fresh weight and **(B)** the survival rate of *S. miltiorrhiza.* PCK, control group; PA, *T. brevicompactum* treatment group. **p < 0.01.

As shown in **Figure 5**, the fresh weight of individual plants in the PA group (108.90 g) was significantly increased, by 17.79%, compared with that in the PCK group (92.45 g; *p*< 0.05). The survival rate in the PA group (80.95%) also increased compared with that in the PCK group (71.43%; no significant difference).

**Figure 6:**
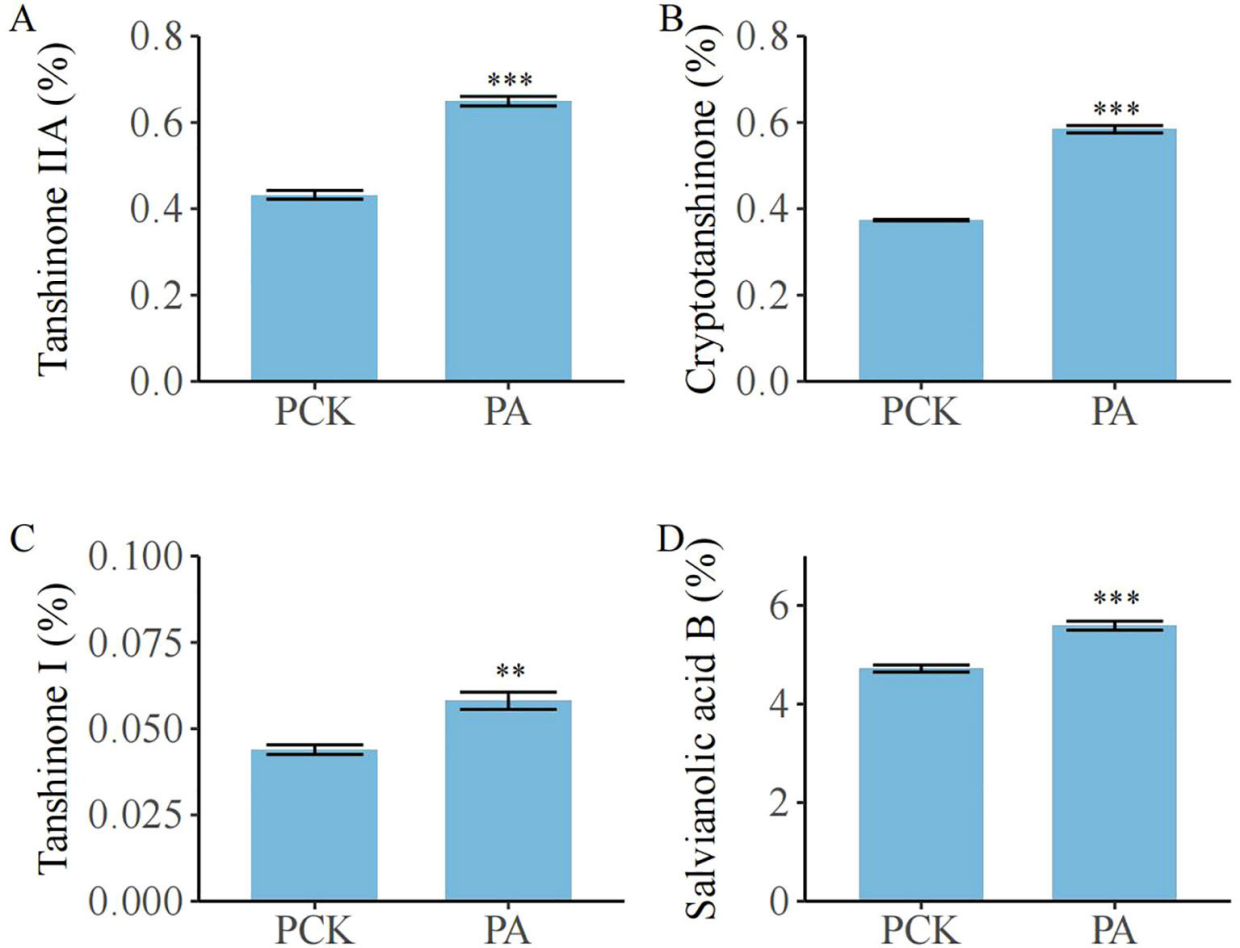
The effect of *T. brevicompactum* on the content of active components in *S. miltiorrhiza* root. **(A)** Tanshinone IIA, **(B)** cryptotanshinone, **(C)** tanshinone I, **(D)** salvianolic acid (B) PCK, control group; PA, *T. brevicompactum* treatment group. **p < 0.01, ***p < 0.001.

There were significant variations in the contents of bioactive ingredients in *S. miltiorrhiza* roots among the treatment groups (**Figure 6**; *p*< 0.05). Compared with the PCK group, the PA group showed a higher content of tanshinone IIA (0.65% *vs*. 0.43%, 51.16% higher than in the PCK group), cryptotanshinone (0.58% *vs*. 0.37%, 56.76% higher than in the PCK group), tanshinone I (0.06% *vs*. 0.04%, 50.00% higher than in the PCK group), and salvianolic acid B (5.59% *vs*. 4.72%, 18.43% higher than in the PCK group).

## 4 Discussion

A previous study has demonstrated that *Trichoderma* has a positive impact on soil physical and chemical properties (Mao and Jiang, 2021). In the present study, the application of *Trichoderma* resulted in a decrease in soil pH and an increase in available nutrient content in the soil. This may be attributable to *Trichoderma* increasing soil nutrient solubilization (Yedidia et al., 2001; Harman et al., 2004). *Trichoderma* can secrete a large amount of organic acids, which can lower soil pH, promote the transformation of plant nutrients (N, P, and K) from noneffective to effective forms, and promote plant growth (Zhang et al., 2019). Redundancy analysis revealed that in this work, AP, NO_3_^-^-N, and NH_4_^+^-N were the dominant factors that shaped the soil microbial community. Furthermore, AP content correlated positively with both Proteobacteria and Planctomycetes, taxa that are key contributors to soil nutrient cycles (Arunrat et al., 2022; Hu et al., 2022). We speculate that the application of *Trichoderma* increased the relative abundance of Proteobacteria and Planctomycetes in the soil, increasing the amount of dissolved phosphorus and promoting the resistance and growth of *S. miltiorrhiza*.

Accumulating studies have shown that *Trichoderma* can improve the soil microbial community structure (Zhang et al., 2020; Mao and Jiang, 2021). This is consistent with our findings (**Figure 1**). Actinobacteria are widely distributed in soil; they decompose organic matter, produce antibiotics that inhibit various plant diseases (Lee et al., 2012; Fuentes et al., 2014; Zheng et al., 2020), and exhibit symbiotic nitrogen fixation and phosphorus solubilization effects (Juhnke et al., 1987; Xu et al., 2023). Chloroflexi are involved in the biogeochemical cycling of C, N, and S, and play a positive role in crop growth (Yang et al., 2024a). *Gemmatimonas* are involved in nitrogen metabolism and transformation (Zhang et al., 2024a); can reduce N_2_O in the soil to nitrate (Zou et al., 2023); and improve soil nutrients, including converting insoluble phosphorus into soluble phosphorus and enhancing nutrient absorption. Consistent with this, *Trichoderma* addition increased AP content in the present study (**Table 1**). Additionally, *Gemmatimonas* can induce plant stress resistance and produce antifungal compounds (Zhu et al., 2021), combat plant pathogens, inhibit soil-borne diseases, and promote plant growth.

*Fusarium* has been shown to be the main pathogen that causes root rot in *S. miltiorrhiza*, leading to a decrease in crop yield and quality (Sa et al., 2022). *Fusarium* in fact causes important diseases in many crops, including tomatoes, corn, cotton, and others (Ma et al., 2010; Srinivas et al., 2019; Liu et al., 2021). We suggest that *Trichoderma* can decrease the incidence of *S. miltiorrhiza* diseases by inhibiting the abundance of pathogens such as *Fusarium*, thereby increasing the yield of *S. miltiorrhiza*.

*T. brevicompactum* treatment significantly improved the content of bioactive components in the roots of *S. miltiorrhiza*. The potential mechanisms may include suppression of pathogenic microorganisms by *Trichoderma*, leading to decreased disease incidence and attenuated damage to the *S. miltiorrhiza* root system, thereby indirectly preserving the biosynthesis of its bioactive constituents (Contreras-Cornejo et al., 2014). Moreover, previous studies have shown that *Trichoderma* can activate the jasmonic acid (JA) and salicylic acid (SA) signaling pathways in plant roots (Islam et al., 2023; Peng et al., 2019), while external application of JA and SA stimulates an increase in secondary compounds in various plant species (Tafreshi et al., 2025). Furthermore, *Trichoderma* secretes organic acids that solubilize insoluble phosphorus and potassium in soil (Bononi et al., 2020), improving nutrient availability to facilitate root development (Chen et al., 2006) and secondary metabolism. *Trichoderma* can also synthesize indole-3-acetic acid, which modulates the root development and distribution of secondary metabolites in *S. miltiorrhiza* (Zhang et al., 2023; Contreras-Cornejo et al., 2024).

## 5 Conclusions

Overall, all three strains of *Trichoderma* tested here (*T. brevicompactum*, *T. viridescens*, and *T. velutinum*) can increase the fresh and dry weights of *S. miltiorrhiza*, improve soil nutrients, increase beneficial microbial abundance, and decrease the abundance of harmful microbial species, thereby alleviating the CC obstacles of *S. miltiorrhiza*. In particular, *T. brevicompactum* was superior to the other tested species. It can improve the survival rate and effective component content of continuous cropping *S. miltiorrhiza*, and has the potential for commercial application. *Trichoderma* can reduce the use of chemical pesticides and fertilizers by regulating the soil microecological balance, inhibiting pathogen reproduction, and enhancing soil fertility. These properties align with the demands of green agriculture and is of great significance for advancing sustainable agricultural development. This research offers a new biological control approach to address continuous cropping obstacles in medicinal plants, ensuring both the quality and safety of medicinal materials and maintaining soil health. Looking ahead, this technology is expected to be extended for use in more crops, supporting the development of resource-efficient and eco-friendly agricultural models. It will drive agriculture toward ecological and sustainable transformation, contributing to high-quality agricultural development.

## Data Availability

The datasets presented in this study can be found in online repositories. The names of the repository/repositories and accession number(s) can be found in the article/supplementary material.
